# Using lesion washout volume fraction as a biomarker to improve suspicious breast lesion characterization

**DOI:** 10.1120/jacmp.v16i5.5187

**Published:** 2015-09-08

**Authors:** Jie Huang, Sarah M. Schafer, Gerald R. Aben, Lori A. Hoisington

**Affiliations:** ^1^ Department of Radiology Michigan State University East Lansing MI USA

**Keywords:** breast MRI, DCE‐MRI, breast tumor characterization, washout curve, lesion WO volume fraction

## Abstract

The purpose of this study was to evaluate using lesion washout (WO) volume fraction as a biomarker to improve the characterization of suspicious breast lesions. Study lesions consisted of a total of 60 malignant tumors (BI‐RADS 6) and 62 suspicious lesions (BI‐RADS 4 or 5). The biopsies of these suspicious lesions resulted in a total of 30 malignant tumors and 32 benign lesions, respectively, yielding a 48.4% positive predictive value (PPV) of the biopsies. The mean and standard deviation of the lesion WO volume fraction of these 60 BI‐RADS 6 malignant tumors were first computed to establish a 99% sensitivity threshold value for malignant tumors, and then the biomarker was used to characterize the suspicious lesions. Using the biomarker would characterize all the malignant tumors as malignant, 12 out of the 32 benign lesions as benign, potentially resulting in a 24% improvement rate in the PPV of the biopsies (from 48.4% to 60%) and consequently a 22.5% reduction rate in the false‐positive rate of benign biopsies (from 51.6% to 40%). The lesion WO volume fraction biomarker could improve the computer‐based assessment of breast MRI by increasing the PPV of breast biopsies and reducing the number of unnecessary biopsies without compromising sensitivity.

PACS number: 87.61.Tg, 87.19.Xj

## I. INTRODUCTION

Breast dynamic contrast‐enhanced magnetic resonance imaging (DCE‐MRI) demonstrates high sensitivity for malignant breast tumor detection, but the specificity is relatively low, resulting in many false‐positive diagnoses of suspicious lesions (BI‐RADS assessment of 4 or 5) in clinical practice, and consequently produces a relatively low positive predictive value (PPV) for biopsies (the number of cancers detected divided by the number of biopsies performed).^1^, ^2^, ^3^, ^4^, ^5^, ^6^ In a recent study involving a total of 125 suspicious breast lesions, the breast biopsies resulted in 42 malignant tumors and 83 benign lesions, showing a 33.6% PPV of the biopsies and, consequently, a 66.4% false‐positive rate of benign biopsies (the ratio of biopsied benign lesions to the number of biopsies performed) at the clinic.^(7)^ Most malignant tumors demonstrate an initial enhancement followed by a rapid washout (WO) or plateau curve in the postcontrast signal intensity time courses. The WO curve mainly reflects the hypervascularity associated with tumor angiogenesis essential for tumor growth.^8^, ^9^, ^10^ Although most benign lesions exhibit a slower, but persistent, enhancement (PE) without the WO behavior, suspicious enhancement curves in some areas showing WO behavior are frequently observed in many benign lesions including fibroadenomas and proliferative fibrocystic changes.^1^, ^4^ These suspicious enhancement curves are mainly responsible for many false‐positive diagnoses of suspicious breast lesions in clinical practice.

Kinetic features of the postcontrast signal intensity time courses correlate with microvessel density; the WO curve has the highest microvessel density, followed by the plateau curve and then the PE curve with the lowest microvessel density.^(8)^ The areas of greatest enhancement in malignant tumors show a typical WO curve and correlate with microvessel density.^(9)^ These findings imply that the WO curve may reflect the hypervascularity associated with tumor angiogenesis and, consequently, the lesion WO volume fraction, characterized as the ratio of the total volume of the WO voxels that demonstrate the WO curve within the tumor to the whole tumor volume, may provide a measure to quantify the hypervascularity of the tumor. While benign proliferative breast diseases can also produce the WO curve, the lesion WO volume fraction for benign proliferation should be relatively small in comparison to that for tumor angiogenesis in malignant tumors, considering that tumor angiogenesis is essential to aggressive cancer tumor growth. Our recent study with a small sample of 28 contrast‐enhanced breast lesions demonstrates the potential of using the lesion WO volume fraction as a new biomarker to differentiate benign from malignant lesions.^(11)^ This study, with a more than four times larger sample size, further tests the potential of using this biomarker to improve the characterization of suspicious breast lesions.

## II. MATERIALS AND METHODS

### A. Lesion selection

Over 1089 standard clinical breast MRI examinations at Michigan State University (MSU) Radiology since 2007 were retrospectively reviewed for abnormal contrast‐enhancing breast lesions. The inclusion criteria were: 1) mass‐like enhancement larger than 5 mm, and 2) assigned a BI‐RADS assessment of 4 (suspicious abnormality), 5 (highly suggestive of malignancy), or 6 (known biopsy‐proven malignancy) at the time when their breast MRI examinations were completed. The exclusion criterion was those lesions with BI‐RADS assessment of 4 or 5 that did not go on to biopsy, or lesions without available histopathology reports.

A total of 122 contrast‐enhanced lesions (46 lesions with BI‐RADS assessment of 4, 16 lesions with BI‐RADS assessment of 5, and 60 lesions with BI‐RADS assessment of 6) involving 112 women (ages from 28 to 82 yrs with mean±SD=53.0±12.0 yrs) were identified and subsequently comprised the lesion set for this study. The 60 malignant tumors with BI‐RADS assessment of 6 were used as a training dataset for determining the biomarker threshold, and the 62 suspicious lesions with BI‐RADS assessment of 4 or 5 were used as a testing dataset for evaluating the biomarker. (No one lesion among these 122 lesions appeared in both datasets, and the two datasets were independent of each other.) The biopsies of these 62 suspicious lesions resulted in a total of 30 malignant tumors and 32 benign lesions. The corresponding PPV of the biopsies was 48.4% and, consequently, the false‐positive rate of benign biopsies was 51.6%. The malignant tumors include infiltrating ductal carcinomas and/or DCIS and invasive lobular carcinomas. The benign lesions include fibrocystic changes, fibrosis, and fibroadenoma.

All of these lesions were radiologically diagnosed by board‐certified, experienced diagnostic radiologists in our clinic, and these diagnoses were reached clinically, independent of this study. The diagnoses followed the current standard ACR BI‐RADS MR imaging lexicon, including the evaluation of lesion morphology and kinetic enhancement analysis using CADstream software (Merge CAD, version 4.1.0; Merge Healthcare, Chicago, IL). This study was approved by the University Institutional Review Board at MSU.

### B. MRI acquisition

**Figure 1:**
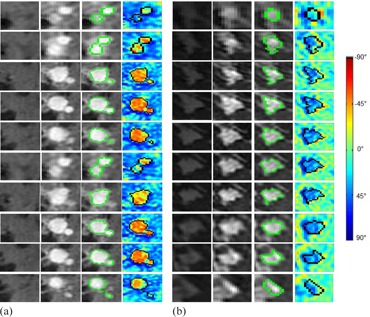
Illustration of lesion ROI and color‐coded kinetic behavior of the postcontrast dynamic period for two representative lesions: (a): a malignant tumor with a WO volume fraction of 69.7% and an estimated lesion size of 1.4 cm; (b): a benign lesion with a WO volume fraction of 11.3% and an estimated lesion size of 1.1 cm. For each lesion, each panel contains four images and each column represents all 10 slices of the lesion. The first two images in each panel show the corresponding precontrast and first postcontrast images. The third image in each panel shows the determined lesion boundary. The last image in each panel shows the corresponding image of the color‐coded angle (degree) of the slope of the fitted straight lines for the postcontrast dynamic period. A negative degree indicates a WO curve.

Bilateral breast imaging was performed on a 1.5 T GE clinical scanner (General Electric Healthcare, Waukesha, WI) using a standard clinical protocol with a dedicated bilateral 8‐channel breast array coil (ASSET: slice acceleration factor 1.0 and phase acceleration factor 3.0). Dynamic images were acquired in the axial plane using a 3D, fat‐suppressed T1‐weighted fast spoiled‐gradient‐echo pulse sequence with the following parameters: TE/TR=2.8/5.9 ms, field of view 320 mm, matrix 320×320, flip angle 10°, slice thickness 2 mm, 116 locations per slab, number of excitations (NEX) 0.76, and ZIP2. Images were reconstructed to an in‐plane resolution of 512×512 pixels, and the application of ZIP2 resulted in the final reconstructed images with a voxel resolution of $0.625\times 0.625\times 1\;{\text{mm}}^{3}$. Gadobenate dimeglumine (Gd‐BOPTA) contrast agent (0.1 mmol/kg) was intravenously injected at a rate of 3 cc/s followed by a 20 cc saline solution flush. One set of precontrast images was acquired immediately prior to the administration of the contrast agent, and the first postcontrast phase was initiated after a 30 s scan delay. Postcontrast imaging included five dynamic phases with a scan time of 1.5 min for each phase, resulted in 7.5 min total scan time. Two representative contrast‐enhanced lesions for the precontrast phase and the first postcontrast phase are shown in Fig. 1.

### C. Lesion WO volume fraction computation

**Figure 2:**
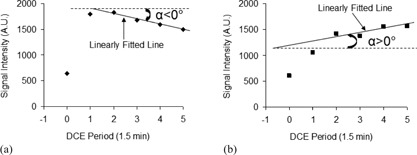
Illustration of linear fitting the signal intensity time course of the postcontrast five phases (1,2,3,4,5) using the least‐squares method: (a): a negative α characterizes a WO curve; (b): a positive α characterizes a PE curve. The angle (α) between the fitted straight line and the horizontal line was computed using α=atan(m)×180/π, where m is the slope of the fitted straight line. AU=arbitrary unit.

An in‐house developed MATLAB‐based (MathWorks, Natick, MA) software algorithm was used to compute lesion WO volume fraction using the same method described in detail in our previous study.^(11)^ This method consists of: 1) determining the boundary of a contrast‐enhanced lesion; 2) quantifying the postcontrast kinetic curve for each voxel within the lesion; 3) defining WO voxels; and then 4) computing the WO volume fraction relative to the whole lesion volume. The boundary of a contrast‐enhanced lesion on the first postcontrast image (phase 1, t=1.5 min) was determined on the difference in the signal intensity between the lesion and its bordering tissue, and then confirmed by two breast MRI diagnostic radiologists independently (Fig. 1). To quantify the overall postcontrast kinetic feature of each voxel, an optimal linear least squares fitting was performed using the last four time points of the postcontrast signal intensity time course of the voxel, and then the slope of the fitted straight line was further computed.^(11)^ A negative slope characterizes a WO curve and a positive slope characterizes a PE curve (Fig. 2). WO voxels were defined as those showing WO curves. Finally, the lesion WO volume fraction was computed as the ratio of the sum of all WO voxels within the lesion to the lesion volume. The lesion boundary determinations and the lesion WO volume fraction computations were blinded to the histopathology reports.

### D. Test for improving the characterization of suspicious breast lesions

To test whether the lesion WO volume fraction can improve the characterization of the suspicious breast lesions, the mean (μ) and standard deviation (σ) of the lesion WO volume fraction of the 60 malignant tumors with BI‐RADS assessment of 6 were used to establish a criterion for characterizing suspicious lesions. Choosing a 99% sensitivity level for detecting malignant tumors yields the threshold value of TH=μ−2.33σ(one‐tail probability<1%) for the lesion WO volume fraction to characterize suspicious lesions. A suspicious lesion is characterized as malignant if the lesion WO volume fraction is equal to or larger than TH, otherwise benign. The comparison of these characterized lesions with their histopathology reports evaluates the effectiveness of the lesion WO volume fraction as a biomarker to improve the characterization of the suspicious breast lesions.

## III. RESULTS

**Figure 3:**
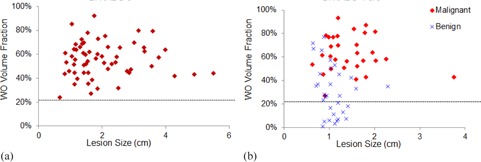
Distribution of the WO volume fraction vs. lesion size. (a): The distribution for the 60 known biopsy‐proven malignant tumors (BI‐RADS assessment of 6). The horizontal dash line indicates the 22.1% threshold value of TH for the selected 99% sensitivity level. (b): The distribution for the 62 suspicious lesions with BI‐RADS assessments of 4 and 5. The lesion types were determined from their histopathology reports. Using the WO volume fraction TH=22.1% as biomarker to characterize these 62 suspicious lesions: 1) all of the 30 malignant tumors would be characterized as malignant, maintaining the same sensitivity; and 2) 12 out of the 32 benign lesions would be characterized as benign, reducing the total number of potentially unnecessary biopsies from 32 to 20.

**Figure 4:**
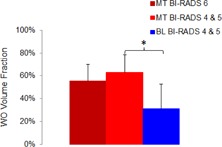
Comparison of WO volume fraction between the malignant tumors and the benign lesions for the three lesion groups. The point indicated by ^*^ represents $p=1.4\times 10^{-8}$. MT=malignant tumor; BL=benign lesion. The error bar denotes the standard deviation.

Figure 3(a) shows the distribution of lesion WO volume fraction versus lesion size for the 60 malignant tumors (BI‐RADS assessment of 6) with μ±σ=55.7%±14.4%. (The lesion size was estimated from the lesion volume using a spherical model.) With the selected 99% sensitivity level, the corresponding threshold value of the lesion WO volume fraction was TH=22.1% and, using this threshold value, all the 60 malignant tumors would be characterize as malignant. Figure 3(b) shows the distribution of lesion WO volume fraction versus lesion size for the 62 suspicious lesions (BI‐RADS assessment of 4 or 5) that included 30 malignant tumors and 32 benign lesions from their histopathology reports. The mean ± standard deviation of the lesion WO volume fraction were 62.9%±15.7% for the 30 malignant tumors, which was slightly larger than that for the 60 malignant tumors with BI‐RADS assessment of 6. These values, however, were 31.2%±21.6% for the 32 benign lesions, significantly smaller than that of the 30 malignant tumors (*t*‐test, $p=1.4\times 10^{-8}$) (Fig. 4). For these 62 suspicious lesions, using the threshold value TH=22.1% would characterize all the malignant tumors as malignant and 12 of the 32 benign lesions as benign. Excluding these 12 characterized benign lesions for biopsy would result in a 24% improvement rate in the PPV of the biopsies (from 48.4% to 60%) and, consequently, a 22.5% reduction rate in the false‐positive rate of benign biopsies (from 51.6% to 40%). Lowering the sensitivity level to 95%, the corresponding threshold value becomes TH=μ−1.65 σ=31.9%(one‐tail probability<5%). For these 62 suspicious lesions, using TH=31.9% would characterize 29 of the malignant tumors as malignant and 17 of the 32 benign lesions as benign. Excluding these 17 characterized benign lesions for biopsy would result in a 36% improvement rate in the PPV of the biopsies (from 48.4% to 65.9%) and, consequently, a 33.9% reduction rate in the false‐positive rate of benign biopsies (from 51.6% to 34.1%).

## IV. DISCUSSION

Breast DCE‐MRI has been shown to be very sensitive for breast malignant tumor detection. The radiological diagnosis of contrast‐enhanced lesions relies heavily on the evaluation of the kinetic behavior of the most enhancing (suspicious) areas of the lesions. The current standard ACR BI‐RADS MR imaging lexicon recommends analyzing the enhancement rate and curve of a lesion by placing a manually selected ROI over the most intensely enhancing area of the lesion.^(12)^ The kinetic WO curve was clearly present in each of the 122 tumors including the 32 benign lesions. For these benign lesions, the presence of the kinetic WO curve was a major factor for their radiological characterization as suspicious lesions, resulting in the potentially unnecessary biopsies.

The significantly larger WO volume fraction for the malignant tumors was probably related to the increased vascularity associated with tumor angiogenesis that is essential for cancer cell growth. The significant difference in the WO volume fraction between the malignant tumors and the benign lesions could be used as an additional MRI parameter for improving the characterization of suspicious contrast‐enhancing breast lesions. With the selected 99% sensitivity level, if the WO volume fraction was used to characterize these suspicious lesions, then the total number of potentially unnecessary biopsies could be reduced by 37.5% (from 32 to 20) and the clinical 48.4% PPV of the biopsies would be improved to 60% PPV. If the sensitivity level is lowered to 95%, then the total number of potentially unnecessary biopsies could be reduced by 53.1% (from 32 to 15) and the clinical 48.4% PPV of the biopsies would be improved to 65.9% PPV.

The lesion size distribution for the 60 malignant tumors (BI‐RADS assessment of 6, Fig. 3(a)) is quite different from that of the 30 malignant tumors from the 62 suspicious lesions (BI‐RADS assessment of 4 or 5, Fig. 3(b)); the former contains 16 lesions with a size larger than 2.5 cm, in comparison to the latter which contains only one lesion with a size larger than 2.5 cm (lesion size=3.75 cm). For the former, the biopsies were performed prior to the breast MRI examinations, showing that all these lesions were under consideration for treatments. For the latter, however, the biopsies were performed after the breast MRI examinations, suggesting that these lesions were either newly diagnosed from screening high‐risk patients or an MRI diagnosis was needed to further evaluate a suspicious lesion from mammogram or ultrasound examinations. Combining all the 90 malignant tumors from the former and the latter together would change μ±σ of the malignant tumor WO volume fraction from 55.7%±14.4% to 58.1%±15.2%. These changed values would change the threshold from 22.1% to 22.7% for the 99% sensitivity level and from 31.9% to 33.0% for the 95% sensitivity level, respectively. With these changed threshold values, however, all testing results of the biomarker would remain the same. These unchanged testing results indicate that the test was not affected by the variation in lesion size distribution between the training and testing datasets.

The spatial distribution of the washout pixels within the lesion varied from tumor to tumor, and no unique spatial distribution was observed in this study. We also did not observe any clear link between the lesion morphology and the spatial distribution of the washout pixels. In comparison to the lesions with a relatively large size (>1 cm), the WO volume fraction appears to be a less sensitive MRI measure for characterizing suspicious contrast‐enhancing breast lesions with a relatively small size (<1 cm). Whether including lesion morphology as a measure can further improve the characterization of small lesions remains to be tested.

In present clinical practice, using CADstream software, a voxel's postcontrast kinetic behavior is visually examined by placing the mouse cursor on the voxel to show its kinetic behavior in a new window, and then moving the cursor around to visually examine the overall kinetic behavior of a manually selected ROI. This kinetic behavior examination is not efficient and convenient, and can be significantly improved with the color‐coded image that shows the overall postcontrast kinetic feature for each voxel (Fig. 1). The corresponding computation is automatic and straightforward, and can be directly implemented in the CADstream software. This color‐coded image enables the examiners to instantly assess the kinetic behavior distribution across the lesion. As it takes substantial time to determine the lesion's boundary on each slice for computing lesion WO volume fraction, this may limit the application of WO volume fraction in daily clinical practice. However, the color‐coded images in Fig. 1 enable the examiners to visually estimate lesion WO volume fraction. The estimated WO volume fraction provides an additional measure that could improve the characterization of suspicious breast lesions in present clinical practice.

In addition to validating our previous study,^(11)^ this retrospective study also aims to establish a justified biomarker threshold for characterizing suspicious lesions. The establishment of this threshold is necessary in order to design a double‐blind prospective study. Using the 60 malignant tumors (BI‐RADS 6) with the determined sensitivity level at 99%, the study establishes the biomarker threshold value of 22.1% for characterizing suspicious lesions. This established biomarker threshold makes it possible to design a double‐blind prospective study that will further test the biomarker and validate the present findings before implementing the biomarker in clinical practice.

## V. CONCLUSIONS

These results show that the WO volume fraction biomarker has potential to improve the computer‐based assessment of breast MRI in clinical practice by increasing the PPV of breast biopsies and reducing the total number of benign biopsies without compromising sensitivity.

## ACKNOWLEDGMENTS

We thank Xuting Zou for screening the patient breast MRI examinations and processing image data. We appreciate MSU Radiology for providing technical support. This work was supported in part by a MSU Targeted Support Grants for Technology Development.

## Supporting information

Supplementary Material

Supplementary Material
