# Supplementary material for: Using lesion washout volume fraction as a biomarker to improve suspicious breast lesion characterization
Source: J Appl Clin Med Phys. 2015 Sep 8;16(5):389–95. doi: 10.1120/jacmp.v16i5.5187 (PMC5690164; doi:10.1120/jacmp.v16i5.5187)
Supplement: Supplementary file 1 — Supplementary Material [file ACM2-16-389-s001.docx]

**Authors’ responses to MRM Referees’ comments, highlighted by underlines**:

Referee: 1

Comments to the Author

The work presented by the authors covers a potentially important topic for breast MRI - the optimum characterisation of dynamic curves and subsequent use to aid diagnosis.

Major points

1. The content of this paper is an extension of a previous Medical Physics paper (reference 11). There does not appear to be new methods involved in the current manuscript, but more patients. In a sense this is therefore a preliminary radiological validation paper (although see below), without much in the way of novel insights into dynamic MRI methods. This suggests that a more radiology/clinically oriented journal may be more suitable than MRM for this work.

The Referee is correct. Our previous study with a small sample of 28 contrast-enhanced breast lesions demonstrated the potential of using the lesion washout volume fraction as a new biomarker to differentiate benign from malignant lesions. With a more than 4 times larger sample size (a total of 122 lesions), the present study (1) validates the previous study and (2) establishes a justified threshold value of the biomarker for improving the characterization of suspicious breast lesions.

2. The authors have applied their methods in a group of patients and taken this opportunity to define a possible threshold to use for identification of malignant lesions. They suggest that this threshold will generate fewer false positive diagnoses using breast MRI. However, the figures quoted cannot be used for further implementation without verification of the proposed threshold in a new, unseen data set. The lack of a separate validation data set is a major weakness in this work.

Besides the 28 lesions in our previous preliminary study, the present study includes a total of 94 new, unseen lesions. The present study is not a simple replication of the previous study, though it indeed validates that study. This study uses a total of 60 malignant tumors (BI-RADS 6) to determine the biomarker threshold value with a 99% sensitivity level for characterizing malignant tumors, and then applies the biomarker to 62 suspicious lesions (BI-RADS 4 or 5) to test the potential value of the biomarker in improving the characterization of suspicious lesions. The study shows that, if the biomarker is used in characterizing these 62 suspicious lesions, without compromising sensitivity, then the biomarker will result in a 24% improvement rate in the PPV of the biopsies and consequently a 22.5% reduction rate in the false-positive rate of the benign biopsies. Accordingly, this is a new and independent study. Although we agree that the implementation of the biomarker requires further verification, hopefully from studies independent of ours, we do not see how “the lack of a separate validation data set is a major weakness in this work”. Prior to any consideration of implementing the biomarker in clinical practice, a double-blind prospective study is required to compare the biomarker with the current clinical practice. But, without first establishing a justified biomarker threshold, it is not possible to design an implementable double-blind prospective study because such a study requires a prior established biomarker threshold. This study achieves that goal. The established biomarker threshold makes it possible to design a double-blind prospective study that is implementable. A brief discussion of the need for a double-blind prospective study to verify our findings is included at the end of the discussion section.

Minor points

1. The use of a tracer kinetic model would characterise the whole wash-in and wash-out curve. It is possible that parameters (e.g. ktrans, ve, etc) derived from such an approach could be at least as effective as the proposed wash-out analysis. The authors should add a discussion of this point in the discussion section.

In our previous Medical Physics paper, we have discussed different kinetic models in the 7^th^ paragraph in the discussion section. It is possible that parameters derived from a tracer kinetic model could be as effective as ours, but we have not seen such a study in the literature. We are not certain a tracer kinetic model could be **at least** as effective as ours, though it is possible.

2. Second paragraph of the introduction: the statement that '...the WO curve has the highest micro vessel density...' makes no sense. Do the authors mean to say that lesions demonstrating a strong wash-out rate have the highest microvessel density?

No, we do not mean that. For a contrast-enhanced tumor, the kinetic behavior of post-contrast signal intensity time course is heterogeneous and varies from area to area within the tumor. The previous two studies (Refs. 8 and 9) showed that, within a tumor, the areas with the WO curve had the highest microvessel density, and the areas with the PE curve had the lowest microvessel density, suggesting that the WO curve may reflect the hypervascularity associated with tumor angiogenesis. Consequently, the lesion WO volume fraction, characterized as the ratio of the total volume of the WO voxels that demonstrate the WO curve within the tumor to the whole tumor volume, may provide a measure to quantify the hypervascularity of the tumor. While benign proliferative breast diseases can also produce the WO curve, the lesion WO volume fraction for benign proliferation should be relatively small in comparison to that for tumor angiogenesis in malignant tumors, considering that tumor angiogenesis is essential to aggressive cancer tumor growth. This is the hypothesis of the new biomarker for differentiating malignant tumors from benign lesions.

3. Methods, part C: it is stated that the last four points are used for the slope fitting. However, figure 2 gives the impression that the last five points are used. Which is correct?

The problem has been corrected.

4. Section V should be titled 'Conclusions'

The mistake has been corrected.

Referee: 2

Comments to the Author

The authors present a study investigating the use of the lesion washout volume fraction as an imaging biomarker for improving the benign / malignant characterization of suspicious breast lesions. They use a group of 60 lesions with a BI-RADS score of 6 to establish a threshold for distinguishing malignant lesions from benign and then apply this to the cases with BI-RADS scores of 4 or 5, resulting in a 22.5% reduction in the false positive rate of benign biopsies at 99% sensitivity. The manuscript is well-presented and clear, although perhaps rather simplistic and limited in scope. I have a number of concerns / queries:

1) I have a conceptual problem with the idea of using the washout volume fraction as a biomarker for detecting malignant from benign lesions, as many malignant lesions exhibit a rim enhancement pattern whereby the outer rim exhibits a typical highly enhancing uptake curve with washout, while the central portion of the lesion exhibits lower enhancement more characteristic of benign or necrotic tissue. Consequently, I would expect these lesions to have relatively low washout volume fraction. Therefore, I feel that the authors should identify those malignant lesions that exhibit a rim enhancing pattern and compare the washout volume fraction to those of more uniformly enhancing appearance. Similarly, the authors make no mention of how the method performs when lesions have poorly-defined edges.

All these questions have been addressed in great detail in our previous Medical Physics paper (see **II.D. Lesion boundary determination**).

2) The comments made by the authors in the last two sentences of the first paragraph of the introduction appear somewhat contradictory. It is suggested that benign lesions exhibit a “slower but persistent enhancement without the WO behavior”, yet “suspicious enhancement curves are frequently observed in many benign lesions”. The definition of a “suspicious enhancement curve” should be clarified – is it one that exhibits washout? Are the authors suggesting that the “false positive diagnosis” occurs because the user typically sparsely samples uptake curves from individual voxels and may by chance just observe those with washout, or that the average uptake curve across the lesion is somehow dominated by those regions that exhibit washout, or something else? Again, this should be clarified and it should be explained why these findings make the choice of lesion washout volume fraction a good one for distinguishing malignant from benign lesions.

To clarify the confusion, we revised the sentence to “Although most benign lesions exhibit a slower but persistent enhancement (PE) without the WO behavior, suspicious enhancement curves in some areas showing WO behavior are frequently observed in many benign lesions including fibroadenomas, proliferative fibrocystic changes, etc.”. All other questions have been addressed in great detail in our previous paper (see the first paragraph in **IV. DISCUSSION AND CONCLUSIONS**).

3) I am interested in how the distinction arises between the BI-RADS 4/5 and BI-RADS 6 assessment scores. Once the BI-RADS 4/5 cases have had a positive biopsy, do they not become a BI-RADS 6 patient? As the study is a retrospective review, is the BI-RADS score that assigned at the time of MRI? This should be clarified. If this is the case, it should also be clarified if there were any differences in the patient (tumor) population that had biopsy before MRI (BI-RADS 6) vs. those that had it after (BI-RADS 4/5).

When a BI-RADS 4/5 case has a positive biopsy, the case becomes a BI-RADS 6, but only after the biopsy. In this retrospective study, the BI-RADS scores were clinically assigned at the time when their MRI exams were completed, independent of this study. We have clarified this in the inclusion criteria under the section A. Lesion Selection. For the 60 lesions with BI-RADS 6, their positive biopsy results were known at the time of MRI. For the 62 lesions with BI-RADS 4/5, their biopsies had not yet been conducted at the time of MRI. Figure 3A shows the distribution of WO volume fraction vs. lesion size for the 60 BI-RADS 6 tumors, and Figure 3B shows the distribution for the 62 BI-RADS 4/5 lesions. Figure 4 shows the comparisons of WO volume fractions for these lesions.

4) Do the authors consider the biopsy to be the “gold standard” in their study? Is it possible that some lesions may have a negative biopsy, but still be malignant?

Yes, we do consider the biopsy to be the gold standard in this study. We do not know the false biopsy rate in clinical practice. Assuming that one or two malignant tumors had false biopsies in this study, by comparing the distributions of WO volume fractions in Figure 3A and 3B, we do not think these few false biopsies would alter the general conclusions of this study.

5) The authors suggest that the least squares fitting was performed on the last four time points, but Fig. 2A, and particularly Fig. 2B, look like the fitting was performed on the last five time points. This should be clarified. Was any optimization performed to determine the best range of points to choose for the fitting?

The problem has been corrected. Yes, in our previous paper we conducted a rigorous test to optimize the choice (see the second paragraph under the section **III.C. Test for improving the characterization of suspicious breast lesions**).

6) The authors neglect to mention a large swathe of the literature that has attempted to provide a more quantitative assessment of lesion enhancement and morphology. If a direct comparison is not provided with the state-of-the-art, then at least some clear justification for why their method is applicable and relevant to current clinical practice should be provided.

In our previous paper, we have discussed different models in the 7^th^ paragraph in the discussion section. We do not think a redundant discussion is necessary, but are willing to do so if the Editor thinks this is necessary.

7) The retrospective nature of the current study is a clear weakness. This should be acknowledged in the discussion / conclusion with recommendation for a future prospective validation of their findings.

This retrospective study aimed to (1) validate our previous study and (2) establish a justified biomarker threshold for characterizing suspicious lesions that can be used to design and implement a future double-blind prospective study to further validate our findings. The aim 2 is a necessity for a double-blind prospective study because it would not be possible to design and implement such a study without a prior established biomarker threshold. It is thus necessary to first carry out this retrospective study and then consider a prospective study. Accordingly, in principle, we do not agree that “The retrospective nature of the current study is a clear weakness”. As suggested, we have added a brief discussion of the need for a double-blind prospective study to verify our findings at the end of the discussion section.

Minor point.

1) Section V. should be labelled “Conclusions”.

The mistake has been corrected.
